# (*E*)-1,2-Bis(4-methyl­phen­yl)ethane-1,2-dione

**DOI:** 10.1107/S1600536808023386

**Published:** 2008-07-31

**Authors:** Hoong-Kun Fun, Reza Kia

**Affiliations:** a X-ray Crystallography Unit, School of Physics, Universiti Sains Malaysia, 11800 USM, Penang, Malaysia

## Abstract

In the mol­ecule of the title compound, C_16_H_14_O_2_, a substituted benzil, the dicarbonyl unit has an *s-trans* conformation. This conformation is substanti­ated by the O—C—C—O torsion angle of 108.16 (15)°. The dihedral angle between the two aromatic rings is 72.00 (6)°. In the crystal structure, neighbouring mol­ecules are linked together by weak inter­molecular C—H⋯O hydrogen bonds and weak inter­molecular C—H⋯π inter­actions. In addition, the crystal structure is further stabilized by inter­molecular π–π inter­actions with centroid–centroid distances in the range 3.6000 (8)–3.8341 (8) Å.

## Related literature

For bond-length data, see Allen *et al.* (1987 ▶). For carbonyl–carbonyl interactions, see Allen *et al.* (1998 ▶). For related structures and applications, see, for example: Fun & Kia, (2008 ▶); Kaftory & Rubin, (1983 ▶); Frey *et al.* (1995 ▶); Crowley *et al.* (1983 ▶); More *et al.* (1987 ▶); Brown *et al.* (1965 ▶); Gabe *et al.* (1981 ▶); Kimura *et al.* (1979 ▶); Stevens & Dubois (1962 ▶); Shimizu & Bartlett, (1976 ▶); Rubin (1978 ▶).
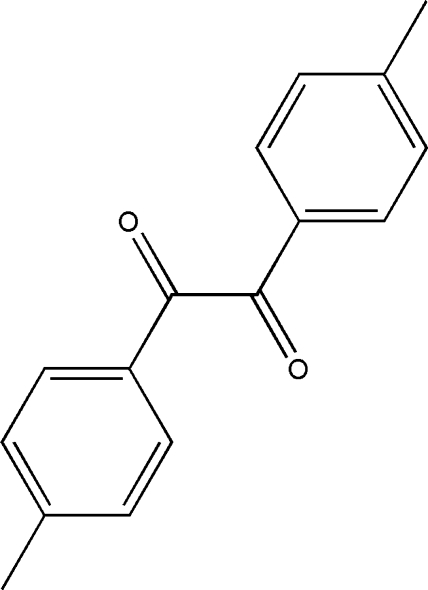


## Experimental

### 

#### Crystal data

C_16_H_14_O_2_
*M* *_r_* = 238.27
Monoclinic, 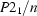

*a* = 6.5658 (1) Å
*b* = 7.0916 (1) Å
*c* = 26.5958 (5) Å
β = 96.473 (1)°
*V* = 1230.46 (3) Å^3^
*Z* = 4
Mo *K*α radiation
μ = 0.08 mm^−1^
*T* = 100.0 (1) K
0.30 × 0.22 × 0.09 mm

#### Data collection

Bruker SMART APEXII CCD area-detector diffractometer
Absorption correction: multi-scan (*SADABS*; Bruker, 2005 ▶) *T*_min_ = 0.975, *T*_max_ = 0.993
15023 measured reflections
3562 independent reflections
2473 reflections with *I* > 2σ(*I*)
*R*_int_ = 0.046

#### Refinement

*R*[*F* ^2^ > 2σ(*F* ^2^)] = 0.052
*wR*(*F* ^2^) = 0.129
*S* = 1.04
3562 reflections
165 parameters
H-atom parameters constrained
Δρ_max_ = 0.34 e Å^−3^
Δρ_min_ = −0.23 e Å^−3^

### 

Data collection: *APEX2* (Bruker, 2005 ▶); cell refinement: *APEX2*; data reduction: *SAINT* (Bruker, 2005 ▶); program(s) used to solve structure: *SHELXTL* (Sheldrick, 2008 ▶); program(s) used to refine structure: *SHELXTL*; molecular graphics: *SHELXTL*; software used to prepare material for publication: *SHELXTL* and *PLATON* (Spek, 2003 ▶).

## Supplementary Material

Crystal structure: contains datablocks global, I. DOI: 10.1107/S1600536808023386/at2603sup1.cif

Structure factors: contains datablocks I. DOI: 10.1107/S1600536808023386/at2603Isup2.hkl

Additional supplementary materials:  crystallographic information; 3D view; checkCIF report

## Figures and Tables

**Table:** *Cg*1 is the centroid of the C1–C6 benzene ring.

C7—C8	1.5350 (19)

**Table:** 

O1⋯O2	3.1702 (15)
*Cg*1⋯*Cg*1^i^	3.6000 (8)
*Cg*1⋯*Cg*1^ii^	3.8341 (8)

Symmetry codes: (i) 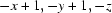; (ii) 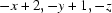.

**Table 2:** Hydrogen-bond geometry (Å, °) *Cg*2 is the centroid of the C9–C14 benzene ring.

*D*—H⋯*A*	*D*—H	H⋯*A*	*D*⋯*A*	*D*—H⋯*A*
C2—H2*A*⋯O1^iii^	0.93	2.44	3.2573 (18)	146
C14—H14*A*⋯*Cg*2^iv^	0.93	2.94	3.6105 (15)	130

Symmetry codes: (iii) 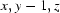; (iv) 
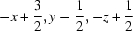
.

**Fig. 1.**
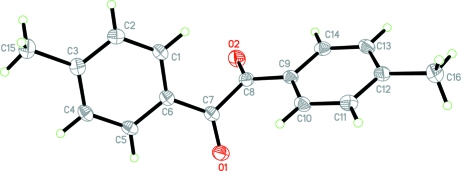
The molecular structure of (I) with atom labels and 50% probability ellipsoids for non-H atoms.

**Fig. 2.**
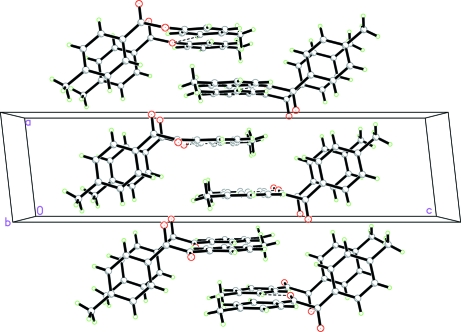
The crystal packing, showing parallel aligning of the benzene rings along the a-axis, and stacking of the molecules down the b-axis. Intermolecular interactions are shown as dashed lines.

## supplementary crystallographic information

### Comment

Investigation of the photophysical properties of the α-dicarbonyls has focused on the intramolecular carbonyl group electronic interaction as a function of their geometrical relationship. As in previous extensive studies of the photochemistry (Stevens & Dubois, 1962; Shimizu & Bartlett, 1976) of these compounds, biacetyl and benzil were the exclusive experimental vehicles for photophysical study. The structure of vicinal di- and polycarbonyl compounds have been of interest for many years (Rubin, 1978; Crowley *et al.*, 1983; Kaftory *et al.*, 1983; Frey *et al.*, 1995; Kimura *et al.*, 1979). Only a limited amount of data has been gathered from solid-state configurations such as in single crystals or as inclusion dopants in host crystals.

In the title compound (I) (Fig.1), bond lengths, bond angles, and torsion angles of the dicarbonyl unit deviate significantly from normal values (Allen *et al.*, 1987) in order to minimize the repulsive interactions resulting from juxtaposition of dipolar carbonyl groups (Allen *et al.*, 1987). The C7–C8 bond distance connecting the carbonyl units is longer than those in normally *sp^2^*–*sp^2^* single bonds, such as in butadiene. This is probably the result of decreasing the unfavourable vicinal dipole-dipole interactions. The dicarbonyl unit has *s-trans* conformation as can be indicated by the torsion angles of O1–C7–C6–C1, and O2–C8–C9–C10 being 169.55 (13) and 179.45 (14)°, respectively. This conformation is substantiated by the torsion angle of O–C–C–O, being 108.16 (15)°. The overal effect is to maximize the distance between the two electronegative oxygen atoms [O1···O2 = 3.1702 (15) Å] and to allow orbital overlap of the dione with the π system of the benzene rings. The dihedral angle between two phenyl rings is 64.74 (5)°. In the crystal structure, neighbouring molecules are linked together by weak intermolecular C—H···O hydrogen bond and weak intermolecular C—H···π interaction. The packing mode (Fig. 2) tend to be dominated by van der Wwaals close packing considerations and the preference for aligning the substituted phenyl rings parallel to each other along the *a* axis at about 3.6000 (8) – 3.8341 (8) Å.

### Experimental

The synthetic method has been described earlier (Frey *et al.*, 1995). Single crystals suitable for *X*-ray diffraction were obtained by evaporation of an methanol solution at room temperature.

### Refinement

All of the hydrogen atoms were positioned geometrically and refined using a riding model with isotropic thermal parameters 1.2 or 1.5 times that of the parent atom.

### Crystal data

**Table:**

C_16_H_14_O_2_	*F*_000_ = 504
*M**_r_* = 238.27	*D*_x_ = 1.286 Mg m^−^^3^
Monoclinic, *P*2_1_/*n*	Mo *K*α radiation λ = 0.71073 Å
Hall symbol: -P 2yn	Cell parameters from 2861 reflections
*a* = 6.5658 (1) Å	θ = 3.0–29.0º
*b* = 7.0916 (1) Å	µ = 0.08 mm^−^^1^
*c* = 26.5958 (5) Å	*T* = 100.0 (1) K
β = 96.473 (1)º	Block, colourless
*V* = 1230.46 (3) Å^3^	0.30 × 0.22 × 0.09 mm
*Z* = 4	

### Data collection

**Table:**

Bruker SMART APEXII CCD area-detector diffractometer	3562 independent reflections
Radiation source: fine-focus sealed tube	2473 reflections with *I* > 2σ(*I*)
Monochromator: graphite	*R*_int_ = 0.046
*T* = 100.0(1) K	θ_max_ = 30.0º
φ and ω scans	θ_min_ = 3.0º
Absorption correction: multi-scan(SADABS; Bruker, 2005)	*h* = −9→9
*T*_min_ = 0.975, *T*_max_ = 0.993	*k* = −9→9
15023 measured reflections	*l* = −37→37

### Refinement

**Table:**

Refinement on *F*^2^	Secondary atom site location: difference Fourier map
Least-squares matrix: full	Hydrogen site location: inferred from neighbouring sites
*R*[*F*^2^ > 2σ(*F*^2^)] = 0.053	H-atom parameters constrained
*wR*(*F*^2^) = 0.129	*w* = 1/[σ^2^(*F*_o_^2^) + (0.0539*P*)^2^ + 0.2688*P*] where *P* = (*F*_o_^2^ + 2*F*_c_^2^)/3
*S* = 1.05	(Δ/σ)_max_ < 0.001
3562 reflections	Δρ_max_ = 0.34 e Å^−^^3^
165 parameters	Δρ_min_ = −0.23 e Å^−^^3^
Primary atom site location: structure-invariant direct methods	Extinction correction: none

### Special details

**Table:**

Experimental. The low-temperature data was collected with the Oxford Cyrosystem Cobra low-temperature attachment.
Geometry. All e.s.d.'s (except the e.s.d. in the dihedral angle between two l.s. planes) are estimated using the full covariance matrix. The cell e.s.d.'s are taken into account individually in the estimation of e.s.d.'s in distances, angles and torsion angles; correlations between e.s.d.'s in cell parameters are only used when they are defined by crystal symmetry. An approximate (isotropic) treatment of cell e.s.d.'s is used for estimating e.s.d.'s involving l.s. planes.
Refinement. Refinement of *F*^2^ against ALL reflections. The weighted *R*-factor *wR* and goodness of fit *S* are based on *F*^2^, conventional *R*-factors *R* are based on *F*, with *F* set to zero for negative *F*^2^. The threshold expression of *F*^2^ > σ(*F*^2^) is used only for calculating *R*-factors(gt) *etc*. and is not relevant to the choice of reflections for refinement. *R*-factors based on *F*^2^ are statistically about twice as large as those based on *F*, and *R*- factors based on ALL data will be even larger.

### Fractional atomic coordinates and isotropic or equivalent isotropic displacement parameters (Å^2^)

**Table:**

	*x*	*y*	*z*	*U*_iso_*/*U*_eq_	
O1	0.78303 (16)	0.85817 (16)	0.10574 (4)	0.0265 (3)	
O2	0.52337 (16)	0.56364 (16)	0.16163 (4)	0.0284 (3)	
C1	0.7563 (2)	0.3603 (2)	0.07576 (5)	0.0216 (3)	
H1A	0.7585	0.3142	0.1086	0.026*	
C2	0.7544 (2)	0.2362 (2)	0.03573 (5)	0.0228 (3)	
H2A	0.7572	0.1071	0.0419	0.027*	
C3	0.7484 (2)	0.3022 (2)	−0.01380 (5)	0.0211 (3)	
C4	0.7468 (2)	0.4963 (2)	−0.02211 (5)	0.0206 (3)	
H4A	0.7422	0.5422	−0.0550	0.025*	
C5	0.7519 (2)	0.6211 (2)	0.01774 (5)	0.0202 (3)	
H5A	0.7533	0.7502	0.0117	0.024*	
C6	0.7550 (2)	0.5541 (2)	0.06734 (5)	0.0193 (3)	
C7	0.7559 (2)	0.6884 (2)	0.10961 (5)	0.0200 (3)	
C8	0.7002 (2)	0.6162 (2)	0.16073 (5)	0.0210 (3)	
C9	0.8525 (2)	0.6278 (2)	0.20558 (5)	0.0197 (3)	
C10	1.0524 (2)	0.6905 (2)	0.20242 (5)	0.0216 (3)	
H10A	1.0934	0.7207	0.1710	0.026*	
C11	1.1896 (2)	0.7079 (2)	0.24546 (5)	0.0234 (3)	
H11A	1.3223	0.7495	0.2428	0.028*	
C12	1.1314 (2)	0.6638 (2)	0.29296 (5)	0.0233 (3)	
C13	0.9340 (2)	0.5956 (2)	0.29583 (5)	0.0237 (3)	
H13A	0.8949	0.5614	0.3271	0.028*	
C14	0.7955 (2)	0.5781 (2)	0.25295 (5)	0.0219 (3)	
H14A	0.6641	0.5331	0.2556	0.026*	
C15	0.7434 (2)	0.1652 (2)	−0.05715 (6)	0.0273 (4)	
H15A	0.7075	0.2309	−0.0885	0.041*	
H15B	0.6436	0.0690	−0.0533	0.041*	
H15C	0.8761	0.1084	−0.0573	0.041*	
C16	1.2805 (3)	0.6890 (3)	0.33977 (6)	0.0316 (4)	
H16A	1.2128	0.6629	0.3692	0.047*	
H16B	1.3302	0.8164	0.3413	0.047*	
H16C	1.3935	0.6038	0.3387	0.047*	

### Atomic displacement parameters (Å^2^)

**Table:**

	*U*^11^	*U*^22^	*U*^33^	*U*^12^	*U*^13^	*U*^23^
O1	0.0327 (6)	0.0224 (6)	0.0251 (5)	−0.0009 (5)	0.0062 (4)	0.0025 (5)
O2	0.0246 (5)	0.0343 (7)	0.0271 (6)	−0.0046 (5)	0.0065 (4)	0.0017 (5)
C1	0.0216 (7)	0.0247 (8)	0.0185 (6)	0.0009 (6)	0.0020 (5)	0.0042 (6)
C2	0.0222 (7)	0.0208 (8)	0.0254 (7)	0.0003 (6)	0.0027 (6)	0.0019 (6)
C3	0.0152 (6)	0.0268 (8)	0.0213 (7)	0.0002 (6)	0.0028 (5)	−0.0007 (6)
C4	0.0160 (6)	0.0282 (8)	0.0178 (6)	0.0001 (6)	0.0020 (5)	0.0043 (6)
C5	0.0171 (6)	0.0221 (8)	0.0215 (7)	0.0012 (6)	0.0028 (5)	0.0054 (6)
C6	0.0157 (6)	0.0233 (8)	0.0189 (6)	0.0003 (6)	0.0027 (5)	0.0021 (6)
C7	0.0171 (6)	0.0230 (8)	0.0202 (7)	0.0010 (6)	0.0030 (5)	0.0031 (6)
C8	0.0245 (7)	0.0186 (8)	0.0205 (6)	−0.0001 (6)	0.0060 (6)	−0.0002 (6)
C9	0.0245 (7)	0.0154 (7)	0.0199 (6)	0.0012 (6)	0.0059 (5)	0.0001 (6)
C10	0.0257 (7)	0.0211 (8)	0.0192 (6)	0.0011 (6)	0.0073 (6)	0.0022 (6)
C11	0.0218 (7)	0.0216 (8)	0.0272 (7)	−0.0003 (6)	0.0041 (6)	0.0024 (6)
C12	0.0308 (8)	0.0161 (7)	0.0226 (7)	0.0027 (6)	0.0007 (6)	0.0013 (6)
C13	0.0339 (8)	0.0189 (8)	0.0195 (7)	0.0026 (7)	0.0081 (6)	0.0017 (6)
C14	0.0242 (7)	0.0191 (8)	0.0237 (7)	−0.0004 (6)	0.0087 (6)	0.0005 (6)
C15	0.0258 (8)	0.0311 (9)	0.0251 (7)	−0.0013 (7)	0.0037 (6)	−0.0040 (7)
C16	0.0381 (9)	0.0292 (9)	0.0261 (8)	−0.0001 (8)	−0.0032 (7)	0.0022 (7)

### Geometric parameters (Å, °)

**Table:**

O1—C7	1.2231 (18)	C9—C14	1.3992 (18)
O2—C8	1.2221 (17)	C10—C11	1.380 (2)
C1—C2	1.380 (2)	C10—H10A	0.9300
C1—C6	1.393 (2)	C11—C12	1.3960 (19)
C1—H1A	0.9300	C11—H11A	0.9300
C2—C3	1.394 (2)	C12—C13	1.393 (2)
C2—H2A	0.9300	C12—C16	1.505 (2)
C3—C4	1.394 (2)	C13—C14	1.382 (2)
C3—C15	1.505 (2)	C13—H13A	0.9300
C4—C5	1.378 (2)	C14—H14A	0.9300
C4—H4A	0.9300	C15—H15A	0.9600
C5—C6	1.3999 (18)	C15—H15B	0.9600
C5—H5A	0.9300	C15—H15C	0.9600
C6—C7	1.473 (2)	C16—H16A	0.9600
C7—C8	1.5350 (19)	C16—H16B	0.9600
C8—C9	1.470 (2)	C16—H16C	0.9600
C9—C10	1.398 (2)		
			
O1···O2	3.1702 (15)	Cg1···Cg1^ii^	3.8341 (8)
Cg1···Cg1^i^	3.6000 (8)		
			
C2—C1—C6	120.39 (13)	C11—C10—C9	120.56 (13)
C2—C1—H1A	119.8	C11—C10—H10A	119.7
C6—C1—H1A	119.8	C9—C10—H10A	119.7
C1—C2—C3	120.77 (15)	C10—C11—C12	120.72 (14)
C1—C2—H2A	119.6	C10—C11—H11A	119.6
C3—C2—H2A	119.6	C12—C11—H11A	119.6
C4—C3—C2	118.69 (13)	C13—C12—C11	118.59 (13)
C4—C3—C15	121.12 (13)	C13—C12—C16	121.20 (13)
C2—C3—C15	120.19 (14)	C11—C12—C16	120.21 (14)
C5—C4—C3	120.87 (13)	C14—C13—C12	121.06 (13)
C5—C4—H4A	119.6	C14—C13—H13A	119.5
C3—C4—H4A	119.6	C12—C13—H13A	119.5
C4—C5—C6	120.22 (14)	C13—C14—C9	120.15 (13)
C4—C5—H5A	119.9	C13—C14—H14A	119.9
C6—C5—H5A	119.9	C9—C14—H14A	119.9
C1—C6—C5	119.05 (13)	C3—C15—H15A	109.5
C1—C6—C7	121.06 (12)	C3—C15—H15B	109.5
C5—C6—C7	119.89 (13)	H15A—C15—H15B	109.5
O1—C7—C6	124.07 (12)	C3—C15—H15C	109.5
O1—C7—C8	117.04 (13)	H15A—C15—H15C	109.5
C6—C7—C8	118.65 (13)	H15B—C15—H15C	109.5
O2—C8—C9	124.19 (12)	C12—C16—H16A	109.5
O2—C8—C7	116.15 (12)	C12—C16—H16B	109.5
C9—C8—C7	119.47 (12)	H16A—C16—H16B	109.5
C10—C9—C14	118.86 (13)	C12—C16—H16C	109.5
C10—C9—C8	121.78 (12)	H16A—C16—H16C	109.5
C14—C9—C8	119.35 (13)	H16B—C16—H16C	109.5
			
C6—C1—C2—C3	−0.9 (2)	O1—C7—C8—C9	−67.01 (18)
C1—C2—C3—C4	0.8 (2)	C6—C7—C8—C9	118.41 (15)
C1—C2—C3—C15	−179.15 (13)	O2—C8—C9—C10	−179.46 (15)
C2—C3—C4—C5	0.3 (2)	C7—C8—C9—C10	−4.7 (2)
C15—C3—C4—C5	−179.83 (13)	O2—C8—C9—C14	−0.6 (2)
C3—C4—C5—C6	−1.2 (2)	C7—C8—C9—C14	174.18 (13)
C2—C1—C6—C5	0.0 (2)	C14—C9—C10—C11	−1.8 (2)
C2—C1—C6—C7	179.66 (13)	C8—C9—C10—C11	177.11 (14)
C4—C5—C6—C1	1.0 (2)	C9—C10—C11—C12	−0.1 (2)
C4—C5—C6—C7	−178.64 (12)	C10—C11—C12—C13	2.2 (2)
C1—C6—C7—O1	169.54 (14)	C10—C11—C12—C16	−178.16 (14)
C5—C6—C7—O1	−10.8 (2)	C11—C12—C13—C14	−2.3 (2)
C1—C6—C7—C8	−16.28 (19)	C16—C12—C13—C14	178.02 (14)
C5—C6—C7—C8	163.39 (12)	C12—C13—C14—C9	0.4 (2)
O1—C7—C8—O2	108.17 (16)	C10—C9—C14—C13	1.6 (2)
C6—C7—C8—O2	−66.42 (18)	C8—C9—C14—C13	−177.28 (13)

Symmetry codes: (i) −*x*+1, −*y*+1, −*z*; (ii) −*x*+2, −*y*+1, −*z*.

### Hydrogen-bond geometry (Å, °)

**Table:**

*D*—H···*A*	*D*—H	H···*A*	*D*···*A*	*D*—H···*A*
C2—H2A···O1^iii^	0.93	2.44	3.2573 (18)	146
C14—H14A···Cg2^iv^	0.93	2.94	3.6105 (15)	130

Symmetry codes: (iii) *x*, *y*−1, *z*; (iv) −*x*+3/2, *y*−1/2, −*z*+1/2.
